# The effect of extracorporeal shock wave therapy on large neurogenic heterotopic ossification in a patient with pontine hemorrhage: A case report and literature review

**DOI:** 10.1097/MD.0000000000031628

**Published:** 2022-10-28

**Authors:** Youngmin Kim, Sook Joung Lee, Eunseok Choi, Sangjee Lee, Jungsoo Lee, Eunjin Park

**Affiliations:** a Department of Physical Medicine and Rehabilitation, College of Medicine, the Catholic University of Korea, Seoul, Republic of Korea.

**Keywords:** bone scan, extracorporeal shock wave therapy (ESWT), function, heterotopic ossification (HO), stroke

## Abstract

Heterotopic ossification (HO), an ectopic bone formation in soft tissue around the joint, is a complication observed in stroke patients. HO around the hip joint causes a reduction in the functional ability of patients by generating pain and limiting range of motion (ROM). In addition, it results in impaired mobility, ultimately affecting quality of life and increasing the mortality of patients. Extracorporeal shock wave therapy (ESWT) has demonstrated efficacy in treating soft tissue inflammation and has been used to reduce patients’ pain in HO. However, almost none of the studies reported degradation in the size of HO on images obtained before and after ESWT application.

**Patient concerns and Diagnosis:**

We report a case of a 36-year-old man who developed HO around both hip joints 3 months after bilateral pontine hemorrhage.

**Interventions:**

Seven months after HO development, ESWT was administered to the area of HO every other day for a total of 10 sessions.

**Outcomes:**

Immediately following treatment, the ROM of both hip joints increased. Thus the patient was able to maintain a sitting posture without having to be bound to the wheelchair. In addition, the tolerable sitting time before groaning increased from less than ten minutes to almost 60 minutes by the end of all ESWT sessions. Unlike other previous reports, a diminished HO size was confirmed by comparing plain X-rays and bone scans obtained before and after treatment sessions.

**Lessons:**

In this case, we report an objective size reduction in HO in radiologic findings after applying ESWT to both hips. ESWT is a safe, easy-to-apply, and noninvasive modality. We would like to emphasize the use of ESWT as a treatment option for HO to decrease the extent of HO, as well as to improve pain, spasticity and function in patients with stroke.

## 1. Introduction

Heterotopic ossification (HO) is characterized by the progressive formation of pathological ectopic bone in soft tissues around the joint.^[1]^ HO associated with disease or injury of the central nervous system can be classified as neurogenic HO (NHO).^[2]^ NHO was reported in 10% to 53% of patients after neurologic injury, mainly in patients with traumatic brain injury or spinal cord injury.^[3]^ The prevalence of HO in stoke patients is known to be 0.5% to 1.2%.^[4]^ HO can occur in both the upper and lower extremities, and among sites, the most common is the hip joint of the paretic limb.^[5]^ HO around the hip joint causes pain and reduces range of motion (ROM), resulting in impairment of mobility, ultimately reducing quality of life and increasing the mortality of patients.^[6]^

Currently, effective and safe methods for treating HO are not clearly established; medications are prescribed mostly for prophylactic purposes, and surgical management burdens patients with a risk of infection or nerve damage.^[1]^ Extracorporeal shock wave therapy (ESWT) is a generator of high-energy acoustic shockwaves, which allows for the initiation of microscopic environmental changes in the tissue where the pulse energy is propagated. It proved to be effective in treating orthopedic disorders, such as plantar fasciitis or lateral epicondylitis.^[7]^ Few studies have applied ESWT for the treatment of HO, and the results have indicated that ESWT was effective in reducing patients’ pain or improving ROM and quality of life.^[5,8–11]^ However, almost none of the studies reported degradation in the size of HO on images obtained before and after ESWT application. Here, we present a case of a 36-year-old male patient with pontine hemorrhage who had severe neurogenic HO on both hip joints. We report an objective size reduction in HO on radiologic findings after applying ESWT to both hips.

## 2. Case report

A 36-year-old man with underlying hypertension became unconscious and quadriplegic when he suffered a spontaneous bilateral pontine hemorrhage. He was immediately admitted to the intensive care unit, where he underwent tracheostomy and received ventilator care for 1 month. Two months later, his level of consciousness returned to an alert state; however, his cognition was still poor, and he was not able to obey commands beyond 1 step. During the intensive care unit period, he was bedridden and could not undergo rehabilitation treatment; as a result, contractures and limitation of range of motion (ROM) developed in multiple joints. Eventually, he was transferred to the general ward without significant improvement in mobility. Three months after stroke onset, computed tomography (CT) and whole-body bone scans were obtained and revealed abundant HO around both hip joints. CT and bone scans revealed that HO was present from the lateral border of the iliac bone all the way down to the proximal portion of the femur and involved ossifying myositis in the vastus muscles. At this point, the patient began receiving rehabilitation treatment, such as tilt table and ROM exercises.

### 2.1. Physical and neurological examinations

It was approximately 10 months after the onset of pontine hemorrhage and 7 months after the first discovery of HO when the patient was admitted to our institution. He displayed circadian rhythms and stayed alert during the day. Although the Mini-Mental-State-Examination score was not accessible, the patient responded to sound by opening and closing the eyes from time to time and made a moaning and groaning sound at pain stimulus, scoring 9 on the revised Coma Recovery Scale. Persistent decerebrated posture was seen in all 4 limbs, and the muscle power of all extremities displayed a Medical Research Council grade of 0-1 (zero to trace). Spasticity was measured to be worse than grade 4 on the Modified Ashworth Scale in both hip flexion and extension and grade 2 to 3 in knee and elbow flexion. Deep tendon reflexes were brisk, and ankle clonus was present in the right leg. Hard and firm HO could be palpated on the lateral side of both hip joints Because of HO on the hip joints, the patient could not use a wheelchair with a natural, proper position. He needed a reclining wheelchair in a fully reclined position with leg support. The patient moaned repeatedly when he stationary in the wheelchair for more than several minutes. His mother, who is his caregiver, claimed that the patient responded in that way when he felt pain. This response was the same as when we applied a noxious stimulus, such as pressing the nailbed hard or scratching the sternum, for motor function evaluation. To improve the patient’s sitting posture and pain, we decided to apply ESWT to the HO around his hip joint.

### 2.2. Intervention

ESWT (MP200, Storz Medical Masterplus®, Tagerwilen, Switzerland) treatment was conducted by a single physiatrist throughout the treatment period. The patient was laid in the supine position. One session of ESWT involved 2000 shocks delivered at a rate of 10 Hz with an energy of 1.2 bar (1 bar = 0.1 MPa = 0.1 N/mm^2^). The treatment was performed on both hips every other day for 10 times in total. Each ESWT session was performed at the same time of day. During the ESWT treatment, there was no change in medication. Additionally, the amount and type of physical therapy (PT) and occupational therapy (OT) before and during the ESWT session were the same. According to the South Korean health insurance standard, PT was performed 2 times per day for 1 hour in total. The first 30 minutes were assigned mainly for the tilt table, and the latter 30 minutes were assigned for ROM exercises provided by a therapist.

During the intervention period, ESWT was administered prior to administration of physical therapy. The passive ROM of both hips was measured immediately after each ESWT session using a standard goniometer, with the patient in either the supine or the decubitus position. The amount of pain sensation was indirectly evaluated by counting the tolerable wheelchair-sitting time before the patient started groaning and moaning loudly. In addition, the serum alkaline phosphatase level, a bone formation marker, was also measured.

### 2.3. Changes in radiologic findings and the patient’s function

**Figure 1.**
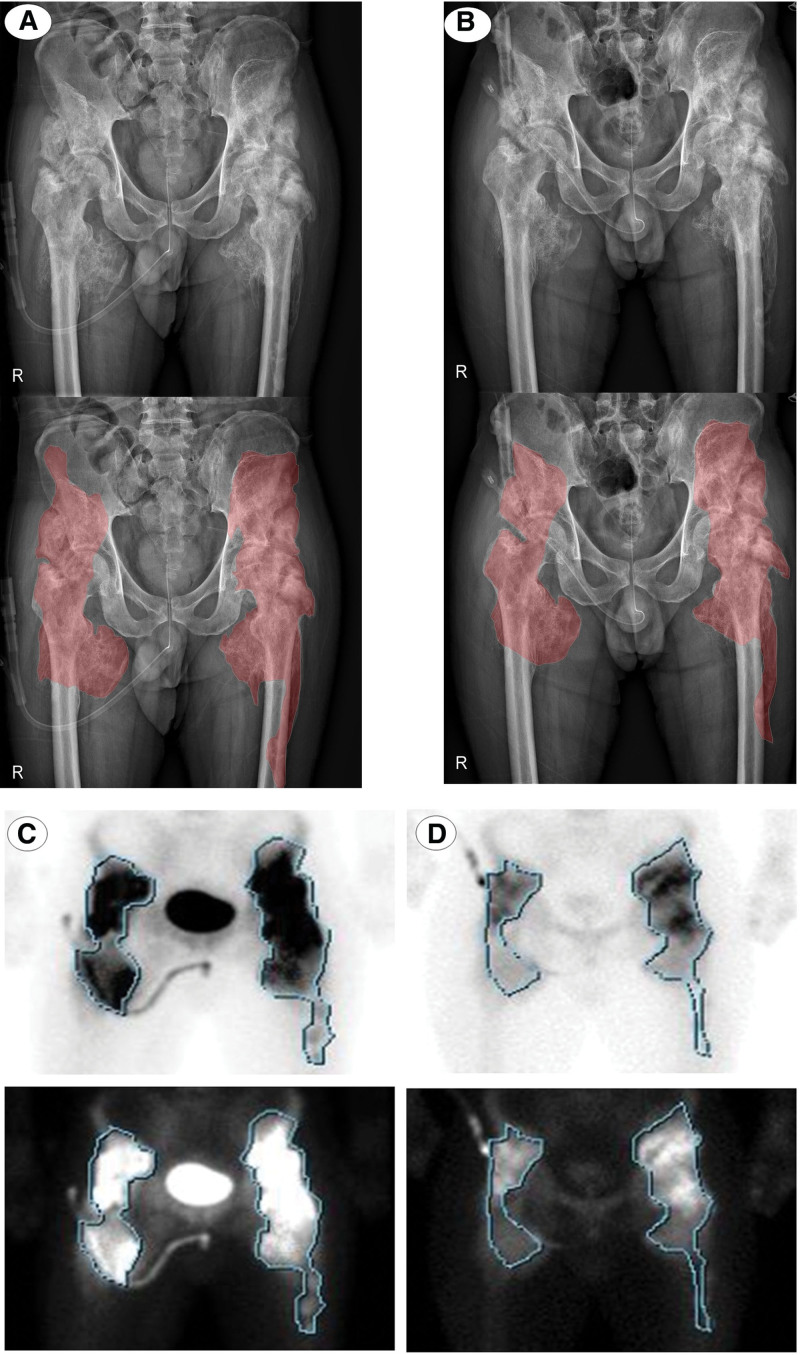
Changes in radiologic findings before and after ESWT treatment: (1-A) Plain X-rays of both hips before (A) and after (B) ESWT (above: raw image; below: with contour of HO); (1-B) Three-phase bone scan before (C) and after (D) ESWT (above: raw image with contour of HO; below: color inverted to show clear boundary of HO). ESWT = extracorporeal shock wave therapy, HO = heterotopic ossification.

**Figure 2.**
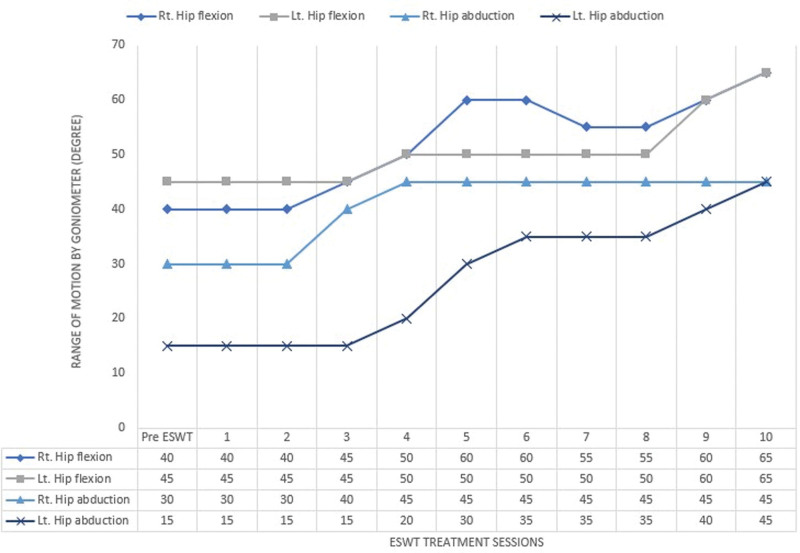
Range of movement of hip flexion and abduction by ESWT treatment session. ESWT = extracorporeal shock wave therapy.

**Figure 3.**
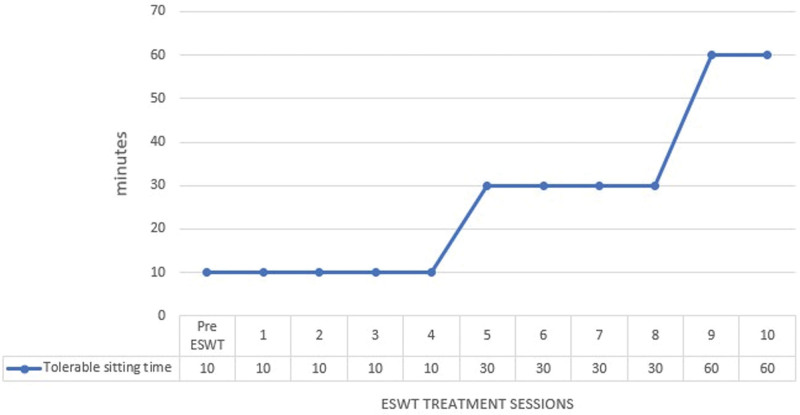
Tolerable sitting time of patients by ESWT treatment session.

**Figure 4.**
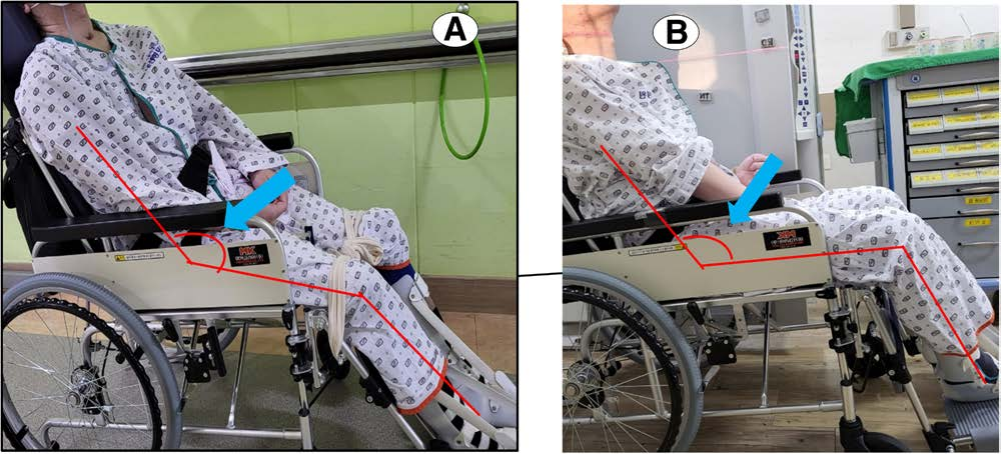
Changes in patient’s sitting posture before and after ESWT treatment: (A) Patient’s sitting posture before ESWT. The rough angle between the upper body and the 2 legs was approximately 135°. (B) Patient’s sitting posture after ESWT. The rough angle between the upper body and both legs is approximately 120° (white arrow: angle between the upper body and both legs). ESWT = extracorporeal shock wave therapy.

Plain hip X-rays and bone scans were obtained before and after the intervention (Fig 1). To evaluate the size of the HO in a 2-dimensional manner on a plain hip X-ray, the contour of the ectopic bone was drawn to measure the estimated area of HO. At the beginning of ESWT, the estimated area of HO was approximately 1740.84 mm^2^ on the right side and 21,182.94 mm^2^ on the left side. When all of the sessions of ESWT were completed, the area of HO on the right side was reduced to 15,062.10 mm^2^, and the area of HO on the left side changed to 18,932.80 mm^2^, proving that the size of the HO was degraded (Fig. 1A). Additionally, when comparing the bone scans obtained at the completion of all treatments with previous images obtained 1 month prior to the intervention, the area with active metabolite uptake around the hip joints, measured by tracing the contour of the lesion, was revealed to have decreased from 1824 mm^2^ to 1254 mm^2^ on the right side and from 2566 mm^2^ to 2107 mm^2^ on the left side (Fig. 1B). The serum alkaline phosphatase level, which was 234 IU/L before treatment, decreased to 177 IU/L after all sessions. After the intervention, the patient’s spasticity on hip flexion and extension slightly improved from MAS grade 4 to 3. Additionally, bilateral hip ROM measured with a goniometer showed gradual improvement as the ESWT sessions continued (Fig. 2). As the ROM increased, the patient was able to maintain a sitting posture without having to be bound to the wheelchair (Figs. 3 and 4). In addition, the tolerable sitting time before groaning increased from less than ten minutes to almost 60 minutes by the end of all ESWT sessions (Figs. 3 and 4). No adverse effect associated with ESWT, such as pain or skin lesions, were not found during and after intervention. Patient’s guardian (Patient’s mother) has provided a written informed consent for inclusion of patient’s clinical and imaging details in the manuscript for the purpose of publication. The case study was approved by the institutional review board of our hospital

## 3. Discussion

HO is a localized and progressive formation of pathological ectopic bone mainly located in the soft tissue around joints.^[5]^ HO around the hip joint causes pain and reduces ROM, resulting in impaired mobility, ultimately reducing the quality of life and increasing the mortality of patients.^[6]^ In this case, we reported a male patient who showed objective reduction in the extent of HO in radiologic findings, as well as functional improvement, after ESWT application on both hips. This finding is significant and suggests that, by applying ESWT to HO, 1 can expect a reduction in the extent of HO, improvement of ROM and better adjusted sitting position.

### 3.1. HO: mechanisms, classification system, and treatment

Although the mechanism of HO formation is not clearly known, the previous literature suggests that multipotent cells in the local tissue constitute a cellular origin of HO.^[1]^ When an inciting event occurs, bone morphogenic protein initiates the secretion of neuroinflammatory factors, such as substance P and calcitonin gene-related peptide, from the sensory nerve.^[12]^ These inflammatory factors stimulate immune cells, such as mast cells, platelets, and neutrophils, and when mast cells are degranulated, they secrete various proteases and matrix metalloproteinases. In turn, these secreted agents stimulate the peripheral nerves to change the activity of the osteoblasts, chondrocytes, and brown adipose tissue that coordinate bone formation in ectopic locations, as well as the creation of new vessels and nerves around newly formed bone.^[13]^

The classification system of HO has varied. Brooker et al classified HO by severity, from the lowest class (class 1), in which an island of bone occurred in the soft tissue around the hip, to the highest class (class 4), involving ankylosis of the hip.^[14]^ The Della Valle classification was simplified into 3 classes.^[15]^ Later, Schmit et al suggested a more practical classification based on the surgical approach, and in this classification, the region and extent of HO were classified separately.^[16]^ In our case, the patient was categorized as class 4 (ankylosis of the hip) in the Brooker classification and class 3 in the Della Valle classification (spurs leaving < 1 cm between opposing surfaces or bony ankylosis), which were the highest levels in each classifying system. In the Schmidt classification, Region II (HOs below and above the tip of the greater trochanter) and Grade C (ankylosis by means of firm bridging from the femur to the pelvis) best describe the patient’s HO.

Currently, methods for treating HO include taking NSAIDS or bisphosphonates or applying radiation, but these methods are only prophylactic and are ineffective when HO has already formed. Physical therapy might improve the ROM and functional ability of patients, but there is controversy regarding whether HO can be aggravated when physical therapy is applied at the acute stage. Operative intervention is an effective method of removing HO all at once, but there is a risk of infection or nerve injury.^[1]^ Accordingly, the need for other methods to treat HO is increasing, and previous studies have also applied ESWT for the treatment of HO.

### 3.2. Mechanisms of ESWT for the treatment of HO: Previous studies (Table 1 )

Extracorporeal shock wave therapy (ESWT) induces microscopic interstitial and extracellular responses in the tissue by generating high-energy acoustic shockwaves and concentrating maximal beneficial pulse energy in the target area.^[7]^ Previous studies have indicated that ESWT was effective in reducing patients’ pain and improving ROM and quality of life in HO (Table 1). In previous studies, the ESWT protocols have varied according to the cause, site and severity of HO. Among cases, the most reported cause of HO was NHO, and the most common site was the hip. The applied ESWT protocol also varied greatly, with 3000 to 4000 shocks applied at frequencies ranging from 3 to 12 Hz and intensities distributed from 1 to 5 bars.^[5,8–11,17–19]^ In this case, the setting of the ESWT was established by referring to the frequency and intensity in the study of the ESWT protocol in the previous study by Li et al, in which there was an actual change in the size of HO on radiological findings. In the study of Li et al, 4000 shocks were applied to the unilateral hip with HO at 8 Hz and energy of 1 bar. In this study, 2000 shocks were applied to each hip so that a total of 4000 shocks were applied. Instead, the frequency and energy intensity were slightly modified to 10 Hz and 1.2, respectively.

**Table 1:** Comparison of previous studies on ESWT applied to HO.

	No. of cases	Etiology (n)	HO site (Severity)	ESWT protocol	Effect	Radiological change
2005, Brissot et al	26	Neurogenic (5), non-neurogenic (21)	1 elbow, 1 wrist, 2 knees, 22 hips (various)	4000 shocks, 3/s) with an energy ranging from 0.54 to 1.06 mJ/mm^2^, once a wk for 4 consecutive wks	Pain (VAS 4.32–>1.14)), Joint flexion (mean increase of 8.18° ± 11.9°), walking distance (from 1126 to 2776 m)	Not mentioned
2013, Reznik et al	1	TBI (1)	Right hip (anterior), (Brooker Class II)	3000 shocks, intensity level of 5–6 bar*, 4 times a wk for 6 wks	Pain (VAS 9->0, 2^nd^ session), hip ROM increased, increased step length	No major change in pre and post hip X-ray
2015, Choi et al	1	TBI (1)	Left hip (Brooker Class III)	4000 shocks, 3 Hz, EFD** 0.056 to 0.068 mJ/mm^2^, once a wk for 3 wks	Pain (VAS 10–>3), 6 min walk test (17–>14 m), sALP (192–>186), WC sitting time (10–>120 min)	No major change in pre and post hip X-ray, increased blood flow in doppler image (US)
2016, Ryu et al	2	SAH (1), hypoxic brain injury (1)	1 Left shoulder, 1 right elbow (Brooker class I/II)	3000 shocks, 12 Hz, intensity level 2–5 bars, 5 times a wk for 4 wks	Pain (NRS 8->0), ROM, muscle strength, hand function improved	Bone scan, X-ray, ultrasound, No major change in images
2017, Reznik et al (ref 8)	11	TBI (11)	7 Hip (3 left 4 right), 4 knee (3 left, 1 right)	3000 shocks, EFD 0.176 mJ/mm^2^ 4 times over 8 wks	ROM, FR (functional reach), MFR (modified functional reach) improved	Not mentioned
2017, Reznik et al (ref 9)	11	TBI (11)	7 Hip (3 left 4 right), 4 knee (3 left, 1 right)	3000 shocks, EFD 0.176 mJ/mm^2^ 4 times over 8 wks	Pain reduction	X-rays, no significant changes
2019, Jeon et al	1	SCI-C4, ASIA A (1)	Right hip (Brooker class I)	4000 shocks, 3Hz, EFD between 0.056–0.068 mJ/mm^2^, 7 times a wk for 7 wks	VAS (7–8–> 3), WC sitting time (less than 1 h–> more than 10 hrs)	No definite change in hip X-rays
2020, Li et al	1	SCI (1)	Left hip (Brooker class IV)	4000 shocks, 8 Hz, intensity level 1 bar, 5 times a wk for 1 year	VAS (8–> 1), ROM improvement, sALP (184–>86)	Ultrasound: size decreased (from 45*25 to 18*16 mm) CT: reduction of ossification mass

ESWT = extracorporeal shock wave therapy, HO = heterotopic ossification, ROM = range of motion, sALP = serum alkaline phosphatase, TBI = traumatic brain injury, VAS = visual analog scale.

*Bar: 1 bar = 0.1 MPa = 0.1 N/mm^2^, **EFD: energy flux density.

The mechanisms of ESWT for musculoskeletal pathologies have been well documented. According to a recently updated review, ESWT promotes the activation of molecular and immunological reactions, improving blood microcirculation, stimulating angiogenesis and increasing neovascularization, activating the anti-inflammatory reaction, and suppressing leukocyte infiltration.^[20]^

In this study, the pain reduction effect of ESWT could only be indirectly assessed by observing diminished moaning and groaning responses and elongated tolerable sitting. However, in previous studies, in which the severity of pain was assessed by the visual analog scale (VAS), a case presented a maximum decrease in the VAS score from 9 to 0. In another study involving 26 patients, the mean VAS score of HO patients decreased from 4.32 to 1.14 after ESWT. Possible mechanisms for the pain relief provided by ESWT treatment were discussed. Shock waves could stimulate nociceptors to send high-frequency nerve impulses, such as in hyperstimulation; thus, the propagation of nerve impulses was blocked according to gate-control theory.^[21]^ ESWT also changes the chemical environment of the cell membrane by generating free radicals, in turn resulting in pain-inhibiting chemicals in the vicinity of the cells. Another possible mechanism is that ESWT inhibits chronic pain by interfering with the neural circuit that promotes chronic pain, reorganizing pathologic memory traces.^[21]^

Although the mechanisms of ESWT action on spasticity due to central nervous system injury are still unknown, variable mechanisms have been proposed. Low-energy ESWT enhances the neuroprotective effect of vascular endothelial growth factor and improves neurological function.^[20]^ ESWT also stimulates the activity of macrophages and Schwann cells, which contribute to the survival and regeneration of neurons.^[20]^ Since the efficacy of ESWT on spasticity is achieved through the reduction of motor neuron excitability, it is expected that application to myotendinous junctions, where the Golgi tendon organ resides, will provide the best outcome.^[22]^ However, in a previous study in which 151 patients were divided into 2 groups, 1 group with ESWT application to the belly muscle and the other to the myotendinous junction, the MAS and Modified Tardieu Scale of both groups decreased after ESWT application without significant difference.^[23]^ Because the patient in our study had large HO on both hips, there was no remarkable change in spasticity.

### 3.3. The effect of ESWT in our case and possible mechanisms for the size reduction of HO

In this case, although the degree of pain could not be measured using a standard scale due to cognitive impairment, the patient’s tolerance of sitting gradually increased after ESWT treatment and was accompanied by improvement in the passive hip flexion angles. An increase in the patient’s tolerance of sitting after ESWT was a meaningful improvement for the patient. Earlier, he would start moaning loudly after only a few minutes and could not receive effective physical therapy or other treatment. Since his tolerance of sitting increased to 1 hour, both the patient’s and caregiver’s quality of life improved since the patient stopped moaning between various tests or physical therapy exercises while waiting and sitting in a wheelchair. Previous studies have also reported that there was a positive behavioral and functional effect, along with reductions in pain and spasticity accompanied by microscopic changes, after ESWT in both hip joints.^[5]^

In addition to reduced pain and improvement in function, the patient showed a marked size reduction in the extent of HO after ESWT on X-ray and bone scans, confirmed by a radiologist and a nuclear medicine physician. For HO to be degraded without surgical removal, it is speculated that changes such as a reduction in angiogenesis and calcium production, fragmentation of calcified deposits by shockwave pressure, and absorbance of ectopic bone to surrounding tissue by phagocytosis should occur.^[19,24]^ Previous reports have scarcely mentioned radiologic changes in HO following ESWT. There was only 1 article depicting a meaningful radiological improvement due to ESWT, but the treatment period was almost an entire year.^[19]^ A major underlying condition that the article and our case have in common is the degree of severity of the patient’s HO, which was graded as Class IV by the Brooker classification.

HO stage and site also affected the outcome of ESWT treatment. Three-phase bone scans are the most sensitive for the early detection of HO since they appear on bone scans 1 to 4 weeks prior to being detected on plain X-rays. The intensity of metabolic activity in the bone scan peaks within a few months after HO formation and decreases after 6 to 12 months, but the activity is maintained in an elevated state as the HO matures.^[25]^ Comparing the 2 bone scan images before and after ESWT application, a clear decrease in the metabolic activities and extent of HO was observed (Fig. 1). The difference in the time points at which the 2 images were obtained might have had an effect, but this effect alone is insufficient to explain this large area of reduction. It is inferred that the anti-inflammatory effect of ESWT also contributed to the changes in the bone scans. When comparing the volume by roughly drawing the contour of the part with uptake on the bone scan image, the reduction in the size of the HO is meaningful since it corresponds to that on plain X-ray (Fig. 1). Additionally, the reason for the reduction in the contour of the mass after applying ESWT in this case might be our patient having massive HO that was closer to the surface of the skin; thus, the effect of ESWT would have been greater. Since the large size of the HO and its location close to the surface seemed to have played a role in the reduction in HO size, it is necessary to further investigate which classifications of HO are indications for ESWT.

### 3.4. Limitations

There are a few limitations of this study. Foremost, this study is a preliminary case study, and due to the patient’s poor mental status, we could not evaluate pain using a standard scale. Because the patient was in a bedridden state, dramatic functional improvement was not observed. Regarding the radiologic reduction in HO size, although the opinions of 2 radiologists were obtained, the use of reformatted CT images or artificial intelligence calculations of HO volume would have been a more objective and definite method. Additionally, the bone scan was not as active after the treatment session, and we cannot conclude that the improvement was only related to ESWT since we did not have a control. The expectation is that the activity would decrease over time, and the finding might have been a coincidence. In future studies, it will be necessary to prepare for objective volume measurements and enroll a control group for bone scanning.

## 4. Conclusion

In this case, we reported an objective size reduction in HO on radiologic findings after applying ESWT to both hips. ESWT is a safe, easy-to-apply, and noninvasive modality. We would like to emphasize the use of ESWT as a treatment option for HO to decrease the extent of HO, as well as improve pain, spasticity and function in patients with stroke.

## Author contributions

**Conceptualization:** Sook Joung Lee, Sangjee Lee, Jungsoo Lee.

**Investigation:** Youngmin Kim, Sook Joung Lee, Eunseok Choi, Jungsoo Lee, Eunjin Park.

**Methodology:** Youngmin Kim, Sook Joung Lee, Eunjin Park.

**Project administration:** Youngmin Kim, Sangjee Lee, Eunjin Park.

**Resources:** Sook Joung Lee.

**Software:** Youngmin Kim.

**Supervision:** Sook Joung Lee.

**Validation:** Eunseok Choi, Jungsoo Lee.

**Writing – original draft:** Youngmin Kim, Sook Joung Lee.

**Writing – review & editing:** Sook Joung Lee, Eunseok Choi, Sangjee Lee.

## References

[1] Ranganathan K Loder S Agarwal S. Heterotopic ossification: basic-science principles and clinical correlates. J Bone Joint Surg Am. 2015;97:1101–11. 26135077 10.2106/JBJS.N.01056 PMC6948799

[2] Jensen LL Halar E Little JW. Neurogenic heterotopic ossification. Am J Phys Med. 1987;66:351–63. 3124630

[3] Medina A Shankowsky H Savaryn B. Characterization of heterotopic ossification in burn patients. J Burn Care Res. 2014;35:251–6. 23872497 10.1097/BCR.0b013e3182957768

[4] Cunha DA Camargos S Passos VMA. Heterotopic ossification after stroke: clinical profile and severity of ossification. J Stroke Cerebrovasc Dis. 2019;28:513–20. 30466894 10.1016/j.jstrokecerebrovasdis.2018.10.032

[5] Reznik JE Gordon SJ Barker RN. Extracorporeal shock wave therapy (ESWT) as a treatment for recurrent neurogenic heterotopic ossification (NHO). Brain Inj. 2013;27:242–7. 23384220 10.3109/02699052.2012.729293

[6] Gennarelli TA. Heterotopic ossification. Brain Inj. 1988;2:175–8. 3139117 10.3109/02699058809150942

[7] Wang CJ. Extracorporeal shockwave therapy in musculoskeletal disorders. J Orthop Surg Res. 2012;7:11. 22433113 10.1186/1749-799X-7-11 PMC3342893

[8] Reznik JE Biros E Sacher Y. A preliminary investigation on the effect of extracorporeal shock wave therapy as a treatment for neurogenic heterotopic ossification following traumatic brain injury. Part II: effects on function. Brain Inj. 2017;31:533–41. 28340312 10.1080/02699052.2017.1283060

[9] Reznik JE Biros E Lamont AC. A preliminary investigation on the effect of extracorporeal shock wave therapy as a treatment for neurogenic heterotopic ossification following traumatic brain injury. Part I: effects on pain. Brain Inj. 2017;31:526–32. 28340308 10.1080/02699052.2017.1283059

[10] Ryu BJ Ha KW Lee JY. Radial extracorporeal shock wave therapy for heterotopic ossification. J Phys Ther Sci. 2016;28:701–4. 27064476 10.1589/jpts.28.701 PMC4793037

[11] Jeon HM Lee WJ Chung HS. Extracorporeal shock wave therapy to treat neurogenic heterotopic ossification in a patient with spinal cord injury: a case report. J Spinal Cord Med. 2021;44:627–30. 31242091 10.1080/10790268.2019.1632597 PMC8288140

[12] McCarthy EF Sundaram M. Heterotopic ossification: a review. Skeletal Radiol. 2005;34:609–19. 16132978 10.1007/s00256-005-0958-z

[13] Kan L Kitterman JA Procissi D. CNS demyelination in fibrodysplasia ossificans progressiva. J Neurol. 2012;259:2644–55. 22736080 10.1007/s00415-012-6563-x PMC3894630

[14] Bedi A Zbeda RM Bueno VF. The incidence of heterotopic ossification after hip arthroscopy. Am J Sports Med. 2012;40:854–63. 22268230 10.1177/0363546511434285

[15] Della Valle AG Ruzo PS Pavone V. Heterotopic ossification after total hip arthroplasty: a critical analysis of the Brooker classification and proposal of a simplified rating system. J Arthroplasty. 2002;17:870–5. 12375245 10.1054/arth.2002.34819

[16] Schmidt J Hackenbroch MH. A new classification for heterotopic ossifications in total hip arthroplasty considering the surgical approach. Arch Orthop Trauma Surg. 1996;115:339–43. 8905109 10.1007/BF00420328

[17] Brissot R Lassalle A Vincendeau S. [Treatment of heterotopic ossification by extracorporeal shock wave: 26 patients]. Ann Readapt Med Phys. 2005;48:581–9. 15993976 10.1016/j.annrmp.2005.04.014

[18] Choi YM Hong SH Lee CH. Extracorporeal shock wave therapy for painful chronic neurogenic heterotopic ossification after traumatic brain injury: a case report. Annal Rehabil Med. 2015;39:318–22. 10.5535/arm.2015.39.2.318 PMC4414981 25932431

[19] Li Y Zhu Y Xie Z. Long-term radial extracorporeal shock wave therapy for neurogenic heterotopic ossification after spinal cord injury: a case report. J Spinal Cord Med. 2022;45:476–80. 32397914 10.1080/10790268.2020.1760507 PMC9135417

[20] Dymarek R Ptaszkowski K Ptaszkowska L. Shock waves as a treatment modality for spasticity reduction and recovery improvement in post-stroke adults—current evidence and qualitative systematic review. Clin Interv Aging. 2020;15:9–28. 32021129 10.2147/CIA.S221032 PMC6954086

[21] Wess OJ. A neural model for chronic pain and pain relief by extracorporeal shock wave treatment. Urol Res. 2008;36:327–34. 18839163 10.1007/s00240-008-0156-2

[22] Yang E Lew HL Özçakar L. Recent advances in the treatment of spasticity: extracorporeal shock wave therapy. J Clin Med. 2021;10:4723. 34682846 10.3390/jcm10204723 PMC8539559

[23] Yoon SH Shin MK Choi EJ. Effective site for the application of extracorporeal shock-wave therapy on spasticity in chronic stroke: muscle belly or myotendinous junction. Annal Rehabil Med. 2017;41:547–55. 10.5535/arm.2017.41.4.547 PMC5608661 28971038

[24] Pleiner J Crevenna R Langenberger H. Extracorporeal shockwave treatment is effective in calcific tendonitis of the shoulder. A randomized controlled trial. Wien Klin Wochenschr. 2004;116:536–41. 15471181 10.1007/BF03217707

[25] Shehab D Elgazzar AH Collier BD. Heterotopic ossification. J Nucl Med. 2002;43:346–53. 11884494

